# Progress in Medicine

**Published:** 1900-03-03

**Authors:** 


### Progress in Medicine.

### DISEASES OF THE NERVOUS SYSTEM.

(*Continued from page* 345.)

**The Pathology of Tabes in Relation to General Paralysis of the Insane** was recently discussed by the Pathological Society.[5] In opening the debate, Mott said that the following facts might be adduced in support of the view that tabes and general paralysis are due to the same morbid process affecting different parts of the nervous system—(1) A certain number of cases of tabes begin with mental symptoms, or such symptoms in the form of crises may occur in the course of tabes ; (2) a certain number of cases of general paralysis present tabetic symptoms during life, and post mortem sclerosis of the posterior columns; and (3) a few cases of tabes have been recorded in which well-marked symptoms of general paralysis ensued. The close relationship between these diseases is shown by their etiology in that they follow syphilis. For instance, in 22 cases of juvenile general paralysis definite signs of syphilis were present in 80 per cent., and could not be excluded in the remainder. Similarly the relationship of syphilis to juvenile tabes had been proved in the recorded cases. Again, various clinical phenomena considered characteristic of tabes, such as grey atrophy, ocular paralysis, perforating ulcers, and Charcot joints were to be seen in general paralytics. The most important physical sign in general paralysis, as in tabes, is reflex iridoplegia, and this is met with otherwise only in syphilitic brain disease. The pathological process in the two diseases is identical —viz., primary progressive degeneration of the neuron with secondary sclerosis and inflammatory or sub-inflammatory conditions in the vessels or adjacent membranes due to irritation caused by the products of degeneration, and a formative proliferation of the glia elements. In the case of tabes the degenerative process affects almost exclusively the first afferent system of neurons and the degeneration corresponds with that which could be obtained by cutting the posterior roots, exogenous tracts being affected, endogenous spared. It might be argued that if the disease were a primary degeneration of the neuron why were not changes more obvious in the posterior root ganglion cells. In six cases examined by Mott in only two were chromolytic changes obvious in these cells, and in both of these the peripheral nerves were affected. In all the cases, however, the cells showed excess of pigmentation and some shrinking and irregularity. The spinal cord lesions of general paralysis with tabetic lesions are the same as those found in simple tabes.

Bruce said that if the degeneration in the posterior columns were due to nutritive changes in the posterior root ganglia, one would expect to find degeneration of the nerve fibres arising from the ganglion, not merely after they entered the posterior columns of the cord, but also in their course between the posterior root ganglia and their place of entry into the cord. This was not found. He was therefore of opinion that the degeneration of the intraspinal course of these fibres is due to pressure on the fibres at their point of entrance into the cord. Normally the posterior root fibres undergo a sharp constriction at this point, and this might be increased by, *e.g.*, meningeal thickening—such thickening might be syphilitic or due to other causes.

Buzzard said that his experience was that mental symptoms might and did, though not very frequently, occur in tabes, though he could not call to mind an instance in which tabes had begun with mental symptoms. And certainly a number of cases of general paralysis present tabetic symptoms during life. Syphilis was certainly the most important factor in the production of both tabes and general paralysis. But it had not yet been proved that syphilis is essential for the production of this degenerative process. And certainly a number of cases present a history of a soft sore only. Hence it seemed premature to put the soft sore out of court as a possible source of a toxin capable of causing the degenerative changes in question. The analogy of peripheral neuritis might be used—the lesions are microscopically the same whatever the particular toxin by which they were brought about.

Gowers said that, of the last 100 cases of tabes he had seen, syphilis was certain in 68, probable in 12, and in the remaining 20 per cent. it was possible, and in not one could the disease be excluded by the absence of exposure to risk of its contraction in the ordinary way. He could confirm Buzzard in saying that when the symptoms of general paralysis and tabes were associated those of tabes precede. He could not recall any case in which the paralytic symptoms led the way, and the knee-jerks afterwards became lost. Reflex iridoplegia was occasionally found in cases of constitutional syphilis apart from any other characteristic nervous symptoms. The theory that the degeneration of the posterior columns in tabes was due to meningeal thickening failed to account for the most remarkable fact of tabes—the restriction of the symptoms to function, *e.g.*, loss of sensation to pain with intact sensibility to touch. The question of the identity of tabes and general paralysis as one disease, due to the same cause, acting on cerebral and spinal neurons, is very much a question of words. The fact of blending forms and cases with common symptoms does not create identity of diseases which differ so widely as the cerebral form of general paralysis and the pure form of tabes. The common use of the word disease is connected with a definite aggregation of symptoms rather than with their discerned causation. Syphilis lowers the durability of the nerve elements, so that some other factor induces a premature decay. An average man inherits an equal vitality in all his structures. At the other extreme are individuals deficient in vital endurance in one or other of their structures; such are some cases of idiopathic muscular atrophy, congenital atrophy of the optic nerves, and hereditary ataxy. Syphilis is a toxic agent which impairs the vitality of certain spinal or cerebral neurons.

Savage agreed that tabes and general paralysis bore close resemblances, and syphilis was the most important factor in both. But other toxins than that of syphilis, of alcohol, lead, and that of influenza might lead to the same result. The ataxic patient often had a neurotic history, but this was not the case in general paralysis.

Payne said that parasyphilitic diseases occurred in organs other than the central nervous system, and this showed that syphilis could produce diseases other than those which could be identified by their anatomical characters. Such diseases were diffuse cirrhosis of the liver of infants and granular kidney of children.

Hale White pointed out that while there are two great clinical groups in which the result of syphilis was manifested, viz., tabes and general paralysis, there is a third, in which the first effect is seen in the peripheral nerves, *e.g.*, those in which the first symptoms of tabes is an ocular paralysis, laryngeal paralysis, optic atrophy.

Batten said that in some cases of tabes the muscle spindles might show degenerative changes, even when the nerves passing to them were normal. This showed that degeneration might start at a place remote from the cell. He could confirm Bruce in that the degeneration in tabes started from the point where the posterior nerve roots enter the spinal cord.

Beevor said that though syphilis was a cause of both tabes and general paralysis, and the pathological process, though attacking different neuron systems, was identical, yet there were well-marked differences between the two diseases. Tabes is of very slow development, general paralysis of rapid progress. It is the exception rather than the rule for cases of tabes to end as general paralytics. Again, in the earliest stage, general paralysis do not show, as a rule, tabetic symptoms other than reflex iridoplegia. In general paralysis the cells of the Rolandic motor area are affected, whilst in tabes the degeneration is confined to the sensory side. Hence, until in the great majority of cases of tabes changes in the crebral cortex were discovered, he would not like to consider tabes and general paralysis as one and the same disease, but would rather look on them as occurring in the same person at the same time.

Head said that the disease process at the bottom of tabes is one which picks out different elements in the afferent system, and hence cannot be due to so gross an anatomical lesion as meningitis at the point of entry of the posterior nerve roots. In the process of destruction of the nerve elements paroxysmal outbursts occur which will assume the character of the element undergoing destruction; thus destruction of the elements which subserve pain impressions will tend to be accompanied by hyperalgesia of the superficial structures of the body and a disturbance in the functions of the viscera, which are supplied by the same segments, constituting the well-known visceral crises. Similarly in general paralysis, destruction of the nerve elements of the motor cortex is accompanied by a paroxysmal disturbance known as a convulsive seizure. The crises of general paralysis and tabes are due essentially to the same process, affecting different nerve elements of the central nervous system.

Abrahams said that, granting that the same etiological factor—syphilis—was present in the great majority of cases of tabes and general paralysis, the reasons why, as a rule, either one or the other set of nerve elements should be attacked were unknown. General paralysis was known to particularly attack persons of considerable mental ability, and the strain on the association neurons no doubt made them especially liable to selection by the syphilitic virus. What etiological factor determined the tabetic lesion to the posterior columns was at present unknown.

[5] Brit. Med. Jour., Nov. 25, Dec. 2, 9, and 23.

